# Online Resources Used by Medical Students, a Literature Review

**DOI:** 10.15694/mep.2020.000136.1

**Published:** 2020-06-25

**Authors:** Laura Ryan, Kate Sheehan, Mohd Ikhwan Marion, Joseph Harbison

**Affiliations:** 1 Trinity College Dublin

**Keywords:** online resource, medical students

## Abstract

This article was migrated. The article was marked as recommended.

**Purpose:** To summarise research pertaining to the use of online resources by medical students throughout the course of their studies in a literature review.

**Method:** Twenty studies published between 2003-2017 were identified for inclusion in the review. All reviewed papers reported on medical students use of online resources for their studies, both in preclinical and clinical settings.

**Results:** Of the studies initially identified, twenty studies focusing on medical students were included and reviewed. The online resources, most frequently mentioned were UpToDate (35%); Epocrates (35%); Medscape (25%), Google (25%); PubMed (20%); Micromedex (20%); Wikipedia (15%); PEPID (10%); Dynamed (10%). Fourteen studies linked the use of online resources to their accessibility and reliability. In nine studies students reported that online resource use enhanced clinical management and diagnostic accuracy.

**Conclusion:** Research on the use of online resources by medical students is largely limited to their use in clinical settings. As technology and learning evolve there is an increased need for students to be able to access such resources online and have the required teaching to understand how best to utilise them.

## Background

In addition to lectures, textbooks and tutorials, medical students are increasingly accessing mobile technology and online resources for learning. There is evidence of widespread use of smartphones and iPads in clinical settings to find information ( Peterson *et al.*, 2004). The number of online resources available is growing exponentially, however, not all resources are equally useful for use by medical students ( Khalifian *et al.*, 2013). The purpose of this literature review is to identify what online resources are commonly used by medical students for learning and why they are used.

## Method

We searched PubMed, Europe PMC, Wiley Online Library, ResearchGate amongst others to select relevant research studies for a literature review and summarised our findings. We decided on criteria that were particularly relevant to our literature review and searched for studies that focused on both medical students and their use of online resources. This search was conducted on the 16 ^th^ March 2020. Twenty studies, published between 2003-2017 were identified, included and reviewed that reported on medical students’ use of online resources, both in preclinical and clinical settings.

### Inclusion and exclusion criteria

We limited our search first to studies carried out from 2003 onwards. We then limited our search to academic journals written in English. As we refined our search, we excluded papers that focused on groups outside of medical students and those that failed to mention the use of electronic resources. We included papers with a focus on medical education. Studies included were not limited to online resource use for any particular learning task. Our definition of medical student didn’t include premedical students. In addition to this, our assessment of what an ‘online resource’ includes was open to any programmes, database or application used by the medical student for learning relevant to any health topic.

## Results

Twenty studies, published between 2003-2017 were identified, included and reviewed. A quarter of studies looked at the use of online resources in both preclinical and clinical settings, while 60% looked only at clinical, and the remainder of studies did not specify.

### Participants

Studies looked at the online resource use of medical students at various stages in their degree, with 10% focusing on 2 ^nd^year, 20% on 3 ^rd^ year, and 15% on 4 ^th^ year medical students. The remaining studies surveyed all medical students in a school, so it is unknown what percentage of their results pertain to which year group.

### Online resources

A variety of online resources were mentioned across these papers, the most dominant being UpToDate (35%); Epocrates (35%); Medscape (25%), Google (25%); PubMed (20%); Micromedex (20%); Wikipedia (15%); PEPID (10%); Dynamed (10%). UpToDate, Epocrates and Medscape were perceived to be among the most trustworthy for preclinical and clinical students ( Quant *et al*., 2016). One study examined what databases were used most by students, showing Ovid, New England Medical Journal and The British Medical Journal as the most popularly used ( Lai and Nalliah, 2010). However, another study indicated that no student downloaded either Ovid or PubMed/Medline ( Chatterley and Chojecki, 2010), suggesting that while used, databases are not the dominant resource used by students online. Three studies reported the use of Wikipedia as a commonly used resource ( Twiss-Brooks *et al*., 2017) ( Brennan *et al*., 2014) ( Choi-Lundberg *et al.*, 2016), one of which reported it as the most frequently accessed resource by students despite recognising that it should be approached with caution due to quality of content and reliability ( Brennan *et al*., 2014).

### Use of resources

Studies reported that online resources were used by medical students to access medical knowledge, to enhance clinical management information and diagnostic accuracy, to inform patient care, and to support test taking. Nine studies reported the reason medical students selected online resources as an educational tool was mainly due to their ease of access, convenience, reliability, alignment with curriculum and quality of information ( Peterson *et al.*, 2004) ( Twiss-Brooks *et al*., 2017) ( Khalifian *et al.*, 2013) ( Brennan *et al*., 2014) ( Choi-Lundberg *et al.*, 2016) ( Garrett and Jackson, 2006) ( Aparicio *et al.*, 2012) ( Baudains *et al*., 2013) ( Lau and Kolli, 2017). Fourteen studies reported that medical students frequently used online resources in a clinical setting in order to access clinical management information and inform patient care ( Peterson *et al.*, 2004) ( Boruff and Storie, 2014) ( Twiss-Brooks *et al*., 2017) ( Khalifian *et al.*, 2013) ( Quant *et al*., 2016) ( Brennan *et al*., 2014) ( Garrett and Jackson, 2006) ( Aparicio *et al.*, 2012) ( Baudains *et al.*, 2013) ( Lau and Kolli, 2017) ( Collier *et al.*, 2007) ( Graber, Tompkins and Holland, 2009) ( Lasserre *et al.*, 2010) ( Klinski *et al.*, 2011) ( Nuss *et al.*, 2014). A comparative study of four free online applications reported that Medscape had the highest satisfaction due to its user-friendly, interactive educational content; Epocrates and Micromedex were both considered excellent for information on drugs, but Epocrates was preferred more for its user-friendly, educational content; and although Google was the most frequently used, it was limited in its clinical management information ( Khalifian *et al.*, 2013). One study showed a more personal use of the PDA as it was used for professional reflection, to get commentary, and assessment ( Garrett and Jackson, 2006). Another study reported that only 4% of students used websites and only 5% used software as a preferred resource for the study of anatomy ( Chatterley and Chojecki, 2010). A further study reported that students sometimes used inadequate internet resources which may not be objective or reliable, leaving much for medical education to address ( Templeman, Robinson and McKenna, 2016).

### Effect of online resources on students

Three studies looked at the effect of the use of online resources on students. One showed that the repeated use of online resources increased the students confidence in navigating different journals ( Lai and Nalliah, 2010). Another demonstrated that the removal of a decision support tool from the student, shows no significant deterioration in the student’s improvements attained, suggesting that there is at least short term sustainability of the education effects of using online resources ( Leung GM *et al.*, 2003). The third study found that 90% thought medical apps enhance clinical knowledge and had a positive effect on their education ( Quant *et al*., 2016).

### Accessibility of resources

One study noted that 59.1% of 3rd and 4th year clinical students used mobile devices to access online information more than once a day, in comparison to only 26.8% of 1st and 2nd year students ( Boruff and Storie, 2014). When it came to learning how to use such resources 75.4% were self-taught, 29.8% peer taught, and 10.5% were taught by the library staff. Students thus suggested instruction sessions may be helpful, with 41.9% suggesting small group interactive sessions, 40.3% an online tutorial, and 16.1% lecture style ( Chatterley and Chojecki, 2010). Interestingly, one study reported that females significantly used more internet resources than male students did (86.9% versus 68.5%) ( Baudains *et al.*, 2013).

## Discussion

In this literature review of twenty studies, evaluating the use of online resources by medical students, we found that papers focused on the use of electronic knowledge resources used by medical students in their preclinical and clinical years. The dominant resources used were Epocrates, Medscape, and UpToDate. Studies all shared focus on clinical skill development through online resources with few studies investigating usage of online resources as a learning aid for lectured subjects eg. physiology. Only 15% of studies looked at incorporation of the use of online resources into curriculum, with students suggesting the use of online tutorials, small group learning, and lectures to instruct students how to best utilise such resources ( Quant *et al*., 2016) ( Lai and Nalliah, 2010) ( Chatterley and Chojecki, 2010). Studies also examined factors such as cost of resources, effect of resources on student learning, and the sustainability of such effects. Another significant factor impacting the use of online resources by medical students is their reluctance to use such resources in front of patients due to fears of appearing less competent ( Quant *et al*., 2016).

## Limitations

This literature review focuses only on studies involving medical students and therefore does not represent the current use or needs of students of other health science disciplines. Knowledge resources are continually evolving, and as a result older studies may be less pertinent to today’s use of online resources. A more significant development within this timeframe is the introduction of smartphones to medical learning. Between 2003 and 2017 there was a 460% increase in internet usage as it became more readily accessible ( Internetworldstats.com, 2020). During this time smartphones too became increasingly popular, allowing medical students access to an abundance of material much faster than before ( Statista.com, 2020). Although many of the older studies focus on Personal Digital Assistant usage, their findings are still significant as it was found that there was no major shift in the general usage pattern of smartphones compared to their predecessor, the PDA. All studies examined the use and outcome of online resources in clinical and preclinical settings, failing to examine the use of such resources as a learning aid for non-clinical subjects e.g. biochemistry.

## Take Home Messages

• As online resources become more accessible and abundant, it is critical that their use both in clinical settings, and as a learning aid are examined, as well as the effect of such aid on the student’s confidence, recall ability, and patient engagement.
• There may be a need for studies examining the usage patterns of medical students over the course of their years studying medicine.
• While most studies seem to analyse the use of scholarly resources, it may be beneficial to look at the use of all online resources used over the course of the medicine degree.
• There is scope too, to examine the use of such resources by other health science students, and the integration of the online resource use into curriculum teaching.
• Due to the recent outbreak of COVID-19 the need for students to carry out learning, lectures and examination from home has highlighted the necessity for further exploration of online resources and their use in medical learning.

## Notes On Contributors

**Laura Ryan** is a medical student in Trinity College Dublin, Ireland.

**Kate Sheehan** is a medical student in Trinity College Dublin, Ireland.

**Mohd Ikhwan Marion** is an Assistant Professor in Medical Education Division, Trinity College Dublin, Ireland.

**Joseph Harbison** is the Director of Teaching and Learning (undergraduate), School of Medicine, Trinity College Dublin, Ireland.

## Declarations

The author has declared that there are no conflicts of interest.

## Ethics Statement

Not required as this is a review of the literature.

## External Funding

This article has not had any External Funding
