# Supplementary material for: Online Resources Used by Medical Students, a Literature Review
Source: MedEdPublish (2016). 2020 Jun 25;9:136. [Version 1] doi: 10.15694/mep.2020.000136.1 (PMC10702663; doi:10.15694/mep.2020.000136.1)
Supplement: Peer review history

# Reviewer report: Reviewer response for version 1

**DOI:** 10.21956/mep.20057.r30762

**Published:** 2020-07-12

**Authors:** Natia Jojua

**Affiliations:** 1 European University

This review has been migrated. The reviewer awarded 4 stars out of 5 Very important topic especially during current pandemic situation. Critical questions and data are presented by authors, however I would like to read more deep analysis of the results and recommendations for the teachers and administration. Many questions are raised from the data given by the authors which can be addressed for the future studies.As it is mentioned in the article majority of the students (75.4%) were self-taught how to use online resources, 29.8% peer taught, and 10.5% were taught by the library staff. How to find relevant information and how to do the critical analysis of it is very important skill of the future doctor. How to navigate in the ocean of information available online must be taught by the teachers from the 1st year of medical education.

Reviewer Expertise:

NA

No decision status is available

# Reviewer report: Reviewer response for version 1

**DOI:** 10.21956/mep.20057.r30760

**Published:** 2020-06-29

**Authors:** James Gray

**Affiliations:** 1 University of Sheffield

This review has been migrated. The reviewer awarded 3 stars out of 5 A relevant topic which is hampered by the search strategy as noted by other reviewers. We have previously published work on this topic in MedEdPublish which is excluded from the search but is pertinent to this particular area of study. https://doi.org/10.15694/mep.2020.000012.1I would commend the authors for mentioning the focus on medical students as certainly there is a lack of literature for wider clinical professions and this is something that could be very usefully explored.

Reviewer Expertise:

NA

No decision status is available

# Reviewer report: Reviewer response for version 1

**DOI:** 10.21956/mep.20057.r30763

**Published:** 2020-06-27

**Authors:** Viktor Riklefs

**Affiliations:** 1 Karaganda Medical University

This review has been migrated. The reviewer awarded 3 stars out of 5 The topic is certainly relevant, especially taking into account the recent COVID-19 pandemic, where most of the schools were forced to online learning and most of the library resources became unavailable to students. However, as noted by previous reviewer, it is not quite clear what were the inclusion/exclusion criteria for literature into this review and why the most recent years (2018, 2019) were not included. Most certainly the article is also lacking the logical framework, explaining the reasons why certain resources are used or not used by students. Without the logical framework it is very hard to make the information useful for the readers, whether they are learners, teachers or administrators. For example, why do students prefer to use resources that lack reliability, such as Google or Wikipedia? Are there certain trends that could be considered universal and should be adapted by different medical schools? But rather than answering these questions, the article just gives the references to different views or different authors, without even making an attempt to bring some foundation to the topic, answer some specific research question or show the gap in the current knowledge.At the same time, the article gives a nice overview of some ideas regarding the use of online resources that are explored in the current literature. This could be used by other researchers to elaborate further on the topic or plan their own research. But it could be much more beneficial if certain recommendations were made to either learners, teachers or administrators to make learning experience in their medical schools better.

Reviewer Expertise:

NA

No decision status is available

# Reviewer report: Reviewer response for version 1

**DOI:** 10.21956/mep.20057.r30761

**Published:** 2020-06-26

**Authors:** P Ravi Shankar

**Affiliations:** 1 American International Medical University

This review has been migrated. The reviewer awarded 4 stars out of 5 The article examines the use of online resources by medical students over a period of fifteen years from 2003 to 2017. Is there any reason why the years 2018 and 2019 were not included? Their use of the term ‘online resources’ was very broad and referred to any program, database or application used by medical students for learning relevant to any health topic. As mentioned by the authors from 2003 there has been increasing use of smartphones and many apps have been developed. Wikipedia is a preferred resource by students and the quality of information on the site is improving. Cost may be an important factor influencing student use of a resource and Wikipedia is well-known as a free resource. It will be interesting to know the full list of indexing and abstracting services used by the authors to search for articles. During the last seventeen years some educational resources have become less popular while others have gained in popularity. The authors mention about the reluctance of students to use these devices in front of patients for fear of appearing less competent. In today’s world information is readily available and the challenge is to critically appraise and make sense of the information. Looking things up as and when required may be a safer alternative to relying on human memory, which is often fallible. Patients should be educated to accept their health professionals looking up information as and when required. Medical and other health science students are learning to access information online as the article mentions and this should be encouraged. Their critical appraisal skills should be developed enabling them to access good quality resources online.

Reviewer Expertise:

NA

No decision status is available
